# Association of non-high-density lipoprotein cholesterol-to-high-density lipoprotein cholesterol ratio (NHHR) with gout prevalence: a cross-sectional study

**DOI:** 10.3389/fnut.2024.1480689

**Published:** 2024-10-24

**Authors:** Xia Guo, Haoxuan Chu, Hanchi Xu, Shipeng Wang, Jiahuan He, Yushi Wang

**Affiliations:** Department of Cardiovascular Medicine, The First Hospital of Jilin University, Changchun, China

**Keywords:** NHHR, gout, NHANES, cross-sectional study, HDL-C

## Abstract

**Background:**

The non-high-density lipoprotein cholesterol (non-HDL-C)-to-high-density lipoprotein cholesterol (HDL-C) ratio (NHHR) is a strong lipid marker that has been associated with atherogenic features. This study aimed to investigate the potential association between the NHHR and gout prevalence.

**Methods:**

This study investigated the relationship between the NHHR and gout by analyzing data gathered from the National Health and Nutrition Examination Survey (NHANES), a research study conducted in the United States from 2007 to 2018. All participants in NHANES provided written informed consent prior to participation. The NHHR was calculated as the ratio of non-HDL-C to HDL-C. Total cholesterol (TC) and HDL-C levels were sourced from NHANES laboratory data. Gout was assessed using a questionnaire. Weighted logistic regression analysis, subgroup analysis, and smoothed curve fitting were performed.

**Results:**

This study included 30,482 participants. The fully adjusted models showed that for each unit increase in NHHR in continuous variables, there was a 10% higher likelihood of gout prevalence (OR: 1.10, 95% CI: 1.05, 1.16). Analysis of the NHHR quartiles revealed that patients in the highest quartile had a notably greater probability of developing gout than those in the lowest quartile. (Q4 vs. Q1, OR: 1.34, 95% CI: 1.05, 1.71). Subgroup analyses yielded consistent results across categories, indicating a significant positive association between the NHHR and gout. E-value analysis suggested robustness to unmeasured confounding. Interaction tests showed that the race, education level, marital relationship, poverty-income ratio (PIR), hypertension, smoking habits, estimated glomerular filtration rate (eGFR), lipid-lowering therapy, and diabetes had no discernible effects on this association. The *p*-values for all the interactions were > 0.05. Nevertheless, the relationship between the NHHR and gout was significantly affected by the age and sex of the participants (interaction *p* < 0.05).

**Conclusion:**

Among adults in the United States, elevated NHHR levels are correlated with increased odds of gout prevalence.

## 1 Introduction

Gout is a common form of inflammatory arthritis that is distinguished by the long-term accumulation of urate crystals in and around the joints and is caused by high uric acid levels (1). With improved living standards, its prevalence have increased globally and are significantly affecting human health (2). Gout is often associated with various comorbidities, including hypertension, cardiovascular disease, obesity, hyperlipidemia, and metabolic syndrome (3). Gout remains a significant global health issue, with a prevalence of 0.58–2.89 cases per 1,000 person-years (4).

Recent studies have identified a new composite indicator of atherogenicity, the non-high-density lipoprotein cholesterol (non-HDL-C)-to-high-density lipoprotein cholesterol (HDL-C) ratio (NHHR) (5). The NHHR is an advanced and comprehensive tool used to evaluate atherogenic lipids. It surpasses existing lipid indices in accurately forecasting the likelihood of developing cardiovascular illnesses (6). The NHHR, a recently identified lipoprotein ratio, accounts for the combined effects of HDL-C and non-HDL-C, which overcomes the prior constraint of separately assessing lipids. The relationship and prognostic significance of the NHHR in different medical conditions, including depression, periodontitis, cardiovascular disease, acute stroke, kidney stones, and diabetes mellitus, have been documented (7–12). Hong et al. (11) revealed a connection between NHHR and the occurrence of kidney stones in which every unit increase in the NHHR resulted in a 4% greater chance of acquiring kidney stones. Additionally, each unit increase in the NHHR was associated with a 9% higher probability of kidney stone recurrence (11). Collectively, these studies suggest that NHHR is a useful predictor of metabolism-related diseases (13). Multiple studies have shown a significant association between serum uric acid (SUA) and lipid levels, such as triglycerides (TG), TC, low-density lipoprotein cholesterol (LDL-C), and HDL-C (14–18). One study examined the relationship between common lipoproteins, including HDL-C and total cholesterol (TC), and the occurrence of gout. HDL-C is thought to have a protective effect in individuals with gout, whereas TC has been positively associated with levels of SUA (19, 20). However, the relationship between the NHHR and gout remains unclear.

We conducted a cross-sectional study utilizing the National Health and Nutrition Examination Survey (NHANES) 2007–2018 dataset to examine the relationship between the NHHR and gout prevalence in American adults. Investigating this association may yield significant information on the connection between lipid metabolism and SUA levels, which is crucial for devising efficient therapies to avert negative health consequences. Positioning the NHHR as a new indicator may offer innovative approaches for assessing and managing gout.

## 2 Methods

### 2.1 Study population

In this analysis, data from the NHANES spanning 2007–2018 were used. The NHANES is a comprehensive research initiative designed to assess the physical well-being and dietary conditions of individuals residing in the United States, including adults and children. The program guarantees that all members undergo biennial health and nutritional examinations. All participants in NHANES provided written informed consent prior to participation. The NCHS study Ethics Review Committee approved the study protocol. The data can be publicly accessed through the NHANES website.

**Figure 1:**
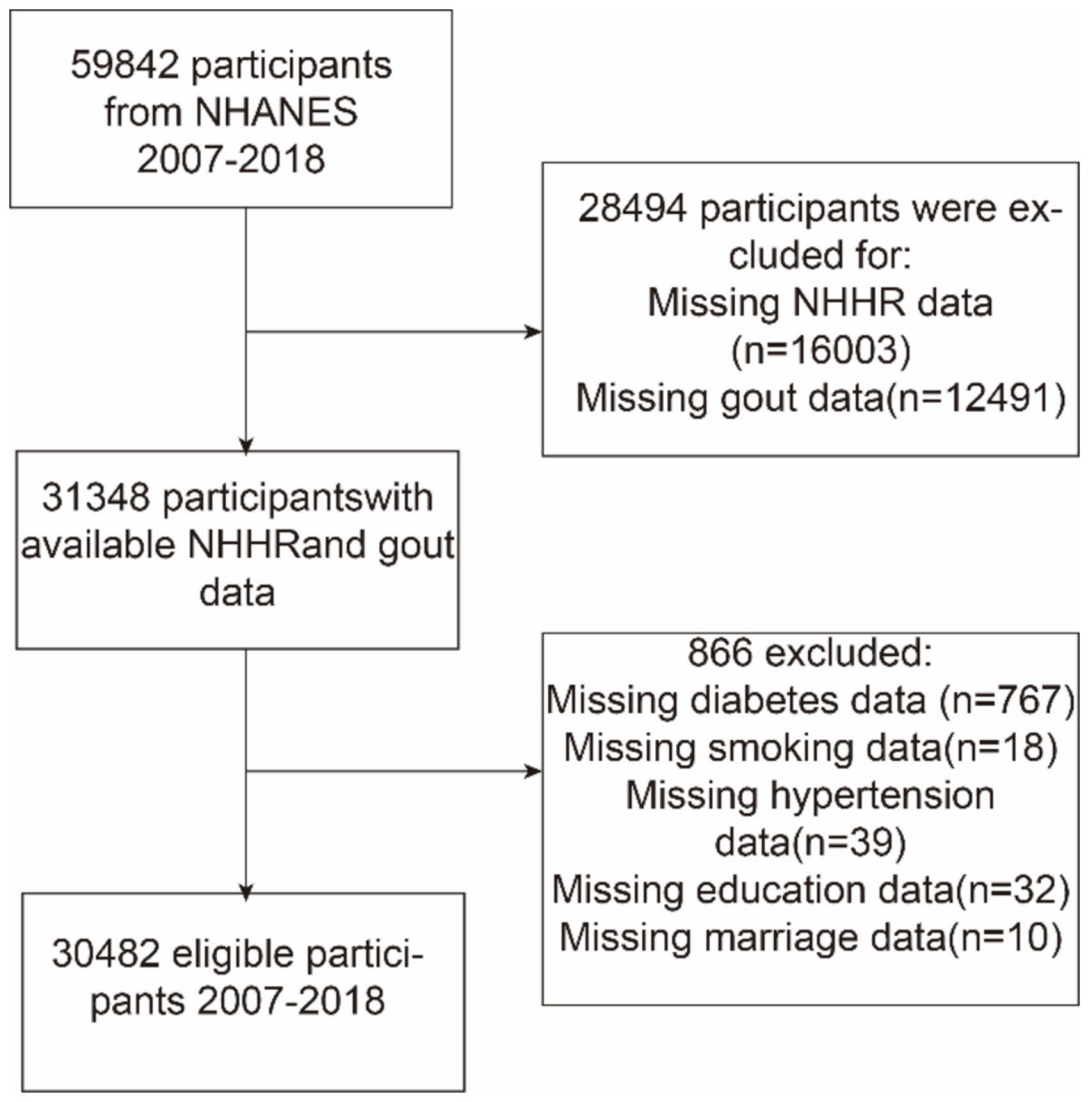
Flowchart depicting participant selection from the National Health and Nutrition Examination Survey (NHANES) (2007–2018). NHHR, non-high-density lipoprotein cholesterol-to-high-density lipoprotein cholesterol ratio.

This study utilized NHANES data from 2007 to 2018 and initially included 59,842 participants. Exclusions were made based on certain demographic characteristics: (a) those with missing NHHR data (*n* = 16,003), (b) those who did not provide data on gout (*n* = 12,491), and (c) those with missing data on co-variables, including education level (*n* = 32), marital status (*n* = 10), hypertension (*n* = 39), diabetes (*n* = 767), and smoking status (*n* = 18). Finally, 30,482 individuals were included in the analysis. Figure 1 shows a flowchart of this procedure.

### 2.2 Exposure definition

The NHHR was included as an independent variable to assess exposure and was determined by dividing the non-HDL-C level by the HDL-C level. The non-HDL-C level was calculated by removing the HDL-C from the TC. TC and HDL-C data were obtained from NHANES laboratory data. Individuals were classified into four tiers based on their NHHR values.

### 2.3 Outcome definition

Gout was an outcome indicator in this study and was derived from the NHANES Health Status Questionnaire, which relied on self-reported responses to specific health questions during personal interviews. All participants were asked, “Have you ever received a diagnosis of gout from a doctor or other healthcare professional?” Participants answered either “yes” or “no.” and were categorized as gouty and non-gouty, respectively. Previous studies have demonstrated the accuracy of gout information obtained using self-reported methods.

### 2.4 Covariables

In order to examine the relationship between NHHR and gout, we considered factors that might influence the results and adjusted out analysis for the following variables: sex, age, diabetes, race, education level, smoking habits, marital status, poverty-income ratio (PIR), alcohol consumption, hypertension, estimated glomerular filtration rate (eGFR), and lipid-lowering therapy. Individuals with high blood pressure were identified as those who responded affirmatively when asked, “Have you ever been told you have high blood pressure?” Similarly, individuals with diabetes were identified as those who answered “yes” to the question, “Has a doctor ever told you that you have diabetes?” Smoking status was ascertained using the question, “Have you consumed a minimum of 100 cigarettes in your lifetime?” Alcohol use was assessed based on an “intake of at least four alcoholic beverages per day.” The three PIR levels were “less than 1,” “1–3,” and “3 or more.” The eGFR was calculated using parameters tailored for each sex. For males, eGFR was calculated as (140 - age) × body weight (kg) × 1.23/serum creatinine (mmol/L). For females, the equation was modified to account for physiological differences, as follows: eGFR = (140 - age) × body weight (kg) × 1.03/serum creatinine (mmol/L) (21). Participants who affirmed the use of prescription medications for cholesterol management during the interview were classified as undergoing lipid-lowering therapy. In this study, we employed various methods to handle missing data for covariates, depending on the proportion of missing values and the type of variable. For covariates with a significant proportion of missing data, such as lipid-lowering therapy (75.5% missing), dummy variables were used to indicate missing covariate values. For covariates with fewer missing values, we used median interpolation to retain as much data as possible and reduce bias. Details regarding the number of missing values and the handling methods for each covariate can be found in Supplementary Table S1.

### 2.5 Statistical analysis

Appropriate statistical weights were applied to adjust the NHANES design. Continuous variables are described as mean ± standard deviation (SD), and categorical variables are presented as percentages. In the categorical model, the NHHR was categorized into quartiles, with the lowest quartile (Q1) serving as the reference group. The relationship between the NHHR and gout was analyzed using multivariable logistic regression models. The NHHR, both in continuous and quartile forms, was included as an independent variable in the models to explore its potential relationship with gout. This study investigated the independent relationship between the NHHR and gout using three distinct models. Model 1 did not account for these variables. Model 2 included modifications to account for sex, age, and ethnicity. Model 3 incorporated further modifications for factors, such as relationship status, education level, PIR, drinking habits, smoking status, diabetes, hypertension, eGFR, and lipid-lowering therapy.

Stratified multivariable regression models were used for subgroup analysis. Stratification was based on several factors, including age, sex, race, marital status, education level, PIR, diabetes, hypertension, intake of at least four alcoholic beverages per day, eGFR, lipid-lowering therapy, and smoking status. Potential differences between the populations were explored in depth. Subsequently, a smoothed curve fitting approach was employed to analyze the positive relationship between the NHHR and gout. We conducted a sensitivity analysis to evaluate the potential confounding effect of alcohol consumption on the association between NHHR and gout. In addition, we explored potential unmeasured confounders between NHHR and gout by calculating E-value (22). The E-value quantifies the required magnitude of an unmeasured confounder that could negate the observed association between NHHR and gout. Statistical significance was set at *p*-values <0.05. Statistical analysis was conducted using R version 3.4.3 with EmpowerStats.

All analyses were conducted using the survey weights provided by NHANES, weighted models mean that in each model we applied a weight suggested by the Centers for Disease Control and Prevention to take into account the oversampling of minorities to provide a final unbiased and accurate estimate of effects for the population.

## 3 Results

### 3.1 Baseline characteristics of participants

Table 1 summarizes the key characteristics of the participants selected from the NHANES between 2007 and 2018 and classified based on the NHHR quartile. Quartiles are defined as follows: Q1 (<1.92), Q2 (1.92–2.66), Q3 (2.66–3.63), and Q4 (>3.63). This study included 30,482 individuals with a median age of 48.01 years. Males accounted for 48.44% of the total participants, whereas females accounted for 51.56%. In this study, 1,427 participants (4.68%) reported a history of gout (Table 1). Participants with higher NHHR values were more likely to be female and Mexican American and non-Hispanic White; have attended some college or hold an AA degree, or 9-11th grade; be married; have a PIR greater than 3; have hypertension, and a history of smoking more than 100 cigarettes in their lifetime; and have a higher eGFR, have lipid-lowering therapy (all *p* < 0.05). The NHHR quartiles differed significantly according to age, smoking, sex, race, HDL-C level, TC level, education level, marital status, diabetes, PIR, alcohol consumption, body mass index (BMI), gout, hypertension, eGFR, SUA level, and lipid-lowering therapy (*p* < 0.01).

**Table 1:** Baseline characteristics of the study population by NHHR quartiles.

Characteristic	Total	Q1 (<1.92)	Q2 (1.92–2.66)	Q3 (2.66–3.63)	Q4 (>3.63)	*p*-value
*N*	30,482	7,614	7,627	7,619	7,622	
Age (years)	49.56 ± 17.69	46.89 ± 0.39	47.82 ± 0.33	47.77 ± 0.31	47.01 ± 0.27	0.036
Sex (*n*, %)						<0.001
Male	14,764 (48.44)	2,697 (35.42)	3,206 (42.03)	4,007 (52.59)	4,854 (63.68)	
Female	15,718 (51.56)	4,917 (64.58)	4,421 (57.97)	3,612 (47.41)	2,768 (36.32)	
Race (*n*, %)						<0.001
Mexican American	4,671 (15.32)	834 (10.95)	1,057 (13.86)	1,300 (17.06)	1,480 (19.42)	
Other Hispanic	3,217 (10.55)	609 (8.00)	787 (10.32)	864 (11.34)	957 (12.56)	
Non-Hispanic White	12,600 (41.34)	3,136 (41.19)	3,126 (40.99)	3,111 (40.83)	3,227 (42.34)	
Non-Hispanic Black	6,252 (20.51)	2051 (26.94)	1700 (22.29)	1,433 (18.81)	1,068 (14.01)	
Other races	3,742 (12.28)	984 (12.92)	957 (12.55)	911 (11.96)	890 (11.68)	
Education level (*n*, %)						<0.001
Less than 9th grade	3,243 (10.64)	578 (7.59)	770 (10.10)	879 (11.54)	1,016 (13.33)	
9–11th grade	4,257 (13.97)	960 (12.61)	983 (12.89)	1,083 (14.21)	1,231 (16.15)	
High school graduate/GED	6,944 (22.78)	1,616 (21.22)	1,694 (22.21)	1822 (23.91)	1812 (23.77)	
Some college or AA degree	8,995 (29.51)	2,310 (30.34)	2,292 (30.05)	2,219 (29.12)	2,174 (28.52)	
College graduate or above	7,043 (23.11)	2,150 (28.24)	1888 (24.75)	1,616 (21.21)	1,389 (18.22)	
Marital status (*n*, %)						<0.001
Married	15,683 (51.45)	3,440 (45.18)	3,844 (50.40)	4,128 (54.18)	4,271 (56.04)	
Widowed	2,397 (7.86)	717 (9.42)	663 (8.69)	570 (7.48)	447 (5.86)	
Divorced	3,305 (10.84)	827 (10.86)	801 (10.50)	834 (10.95)	843 (11.06)	
Separated	1,040 (3.41)	267 (3.51)	252 (3.30)	251 (3.29)	270 (3.54)	
Never married	5,584 (18.32)	1755 (23.05)	1,461 (19.16)	1,262 (16.56)	1,106 (14.51)	
Living with partner	2,473 (8.11)	608 (7.99)	606 (7.95)	574 (7.53)	685 (8.99)	
PIR (*n*, %)						<0.001
<1	6,101 (20.02)	1,447 (19.00)	1,492 (19.56)	1,478 (19.40)	1,684 (22.09)	
1–3	14,506 (47.59)	3,439 (45.17)	3,561 (46.69)	3,753 (49.26)	3,753 (49.24)	
≥3	9,875 (32.40)	2,728 (35.83)	2,574 (33.75)	2,388 (31.34)	2,185 (28.67)	
Alcohol use (drinks) (*n*, %)						<0.001
<4	27,567 (90.44)	6,980 (91.67)	7,025 (92.11)	6,876 (90.25)	6,686 (87.72)	
≥4	2,915 (9.56)	634 (8.33)	602 (7.89)	743 (9.75)	936 (12.28)	
BMI (kg/m^2^)	29.18 ± 6.91	26.10 ± 0.09	28.62 ± 0.11	30.13 ± 0.12	31.37 ± 0.11	<0.001
HDL-C (mg/dL)	52.93 ± 16.16	70.38 ± 0.35	56.20 ± 0.22	48.12 ± 0.14	38.87 ± 0.15	<0.001
Total Cholesterol (mg/dL)	192.29 ± 41.83	171.77 ± 0.73	183.50 ± 0.68	196.59 ± 0.61	221.97 ± 0.77	<0.001
NHHR	2.93 ± 1.47	1.48 ± 0.01	2.28 ± 0.00	3.10 ± 0.00	4.85 ± 0.02	<0.001
Diabetes (*n*, %)						0.006
No	26,339 (86.41)	6,632 (87.10)	6,639 (87.05)	6,552 (86.00)	6,516 (85.49)	
Yes	4,143 (13.59)	982 (12.90)	988 (12.95)	1,067 (14.00)	1,106 (14.51)	
Gout (*n*, %)						<0.001
No	29,056 (95.32)	7,304 (95.93)	7,300 (95.71)	7,247 (95.12)	7,205 (94.53)	
Yes	1,426 (4.68)	310 (4.07)	327 (4.29)	372 (4.88)	417 (5.47)	
Smoking habit (*n*, %)						<0.001
No	17,064 (55.98)	4,525 (59.43)	4,485 (58.80)	4,267 (56.00)	3,787 (49.69)	
Yes	13,418 (44.02)	3,089 (40.57)	3,142 (41.20)	3,352 (44.00)	3,835 (50.31)	
Hypertension (*n*, %)						<0.001
No	19,593 (64.28)	5,089 (66.84)	4,905 (64.31)	4,816 (63.21)	4,783 (62.75)	
Yes	10,889 (35.72)	2,525 (33.16)	2,722 (35.69)	2,803 (36.79)	2,839 (37.25)	
eGFR (ml/min/1.73 m^2^)	114.12 ± 47.89	105.13 ± 0.64	114.52 ± 0.80	122.31 ± 0.84	130.81 ± 0.74	<0.001
SUA (mg/dL)	5.45 ± 1.45	4.88 ± 0.02	5.21 ± 0.02	5.57 ± 0.02	6.00 ± 0.03	<0.001
Lipid-lowering therapy (*n*, %)						<0.001
Not recorded	23,024 (75.53)	5,871 (77.11)	5,824 (76.36)	5,741 (75.35)	5,588 (73.31)	
No	1,662 (5.45)	151 (1.98)	242 (3.17)	447 (5.87)	822 (10.78)	
Yes	5,796 (19.01)	1,592 (20.91)	1,561 (20.47)	1,431 (18.78)	1,212 (15.90)	

The quartiles are defined as follows: Q1 (<1.92), Q2 (1.92–2.66), Q3 (2.66–3.63), and Q4 (>3.63). This table presents the baseline characteristics of the study population divided into quartiles of non-high-density lipoprotein cholesterol-to-high-density lipoprotein cholesterol ratio (NHHR). Continuous variables are described as weighted mean ± standard deviation (SD), while categorical variables are presented as unweighted n (%). NHHR, non-high-density lipoprotein cholesterol-to-high-density lipoprotein cholesterol ratio; BMI, body mass index; HDL-C, high-density lipoprotein cholesterol; PIR, poverty-income ratio; eGFR, estimated glomerular filtration rate. SUA: serum uric acid level.

### 3.2 The association between the NHHR and gout

Table 2 illustrates the relationship between the NHHR and gout. The results demonstrated a clear and direct relationship between elevated NHHR and an increased prevalence of gout in continuous models. The link between the NHHR and the odds of gout prevalence was evident, as was the unadjusted baseline model and the fully adjusted model. In the model that accounted for all relevant factors, the chance of gout prevalence increased by 10% for every unit of increase in the NHHR (odds ratio [OR]: 1.10; 95% CI: (1.05, 1.16)). In the categorical model, specifically the partially adjusted model (model 2), the fourth quartile (Q4) exhibited a 62% higher prevalence of gout (OR: 1.62; 95% CI: 1.27, 2.05) compared with the lowest NHHR quartile (Q1). In model 3, the ORs for the probability of gout in the Q2, Q3, and Q4, compared to Q1, were 1.03 (95% CI: 0.82, 1.30), 1.04 (95% CI: 0.81, 1.33), and 1.34 (95% CI: 1.05, 1.71), respectively. To account for the potential confounding effect of alcohol consumption, we performed a sensitivity analysis in the group of participants who did not report alcohol consumption. The overall association between NHHR and gout remained consistent, as shown in Supplementary Table S2. In addition, we generated an E-value (1.43) to assess the sensitivity to unmeasured confounding. The primary findings were robust, unless an unmeasured confounder existed with a lower OR.

**Table 2:** Weighted logistic regression results showing association between NHHR and gout.

	Model 1	Model 2	Model 3
	OR (95%CI), *p*-value	OR (95%CI), *p*-value	OR (95%CI), *p*-value
Exposure			
Continuous	1.13 (1.08, 1.18), <0.001	1.15 (1.10, 1.21), <0.001	1.10 (1.05, 1.16), <0.001
NHHR quartile			
Q1	Reference	Reference	Reference
Q2	1.13 (0.91, 1.40), 0.283	1.08 (0.86, 1.36), 0.498	1.03 (0.82, 1.30), 0.792
Q3	1.21 (0.95, 1.53), 0.120	1.16 (0.90, 1.49), 0.242	1.04 (0.81, 1.33), 0.747
Q4	1.61 (1.29, 2.01), <0.001	1.62 (1.27, 2.05), <0.001	1.34 (1.05, 1.71), 0.023
*p* for trend	<0.001	<0.001	0.031
Male			
Continuous	1.01 (0.95, 1.07), 0.761	1.11 (1.05, 1.17), <0.001	1.07 (1.01, 1.13), 0.037
NHHR quartile			
Q1	Reference	Reference	Reference
Q2	0.83 (0.63, 1.10), 0.198	0.91 (0.69, 1.21), 0.523	0.87 (0.66, 1.14), 0.311
Q3	0.79 (0.58, 1.06), 0.122	0.99 (0.72, 1.34), 0.930	0.89 (0.65, 1.21), 0.465
Q4	0.86 (0.66, 1.12), 0.272	1.26 (0.96, 1.66), 0.103	1.06 (0.80, 1.39), 0.692
*p* for trend	0.377	0.064	0.530
Female			
Continuous	1.26 (1.15, 1.37), <0.001	1.27 (1.16, 1.39), <0.001	1.19 (1.09, 1.30), <0.001
NHHR quartile			
Q1	Reference	Reference	Reference
Q2	1.40 (0.93, 2.11), 0.107	1.38 (0.91, 2.09), 0.133	1.32 (0.86, 2.02), 0.206
Q3	1.46 (1.02, 2.07), 0.039	1.46 (1.02, 2.09), 0.043	1.29 (0.89, 1.86), 0.180
Q4	2.52 (1.75, 3.64), <0.001	2.57 (1.75, 3.76), <0.001	2.05 (1.39, 3.03), <0.001
*p* for trend	<0.001	<0.001	0.001

Model 1: unadjusted. Model 2: Adjusted for sex, age, and race. Model 3: Adjusted for sex, age, race, education level, smoking, drinking, marital status, PIR, diabetes, hypertension, eGFR, and lipid-lowering therapy. The stratified analysis was not adjusted for sex.

### 3.3 Smoothed curve fitting analysis

**Figure 2:**
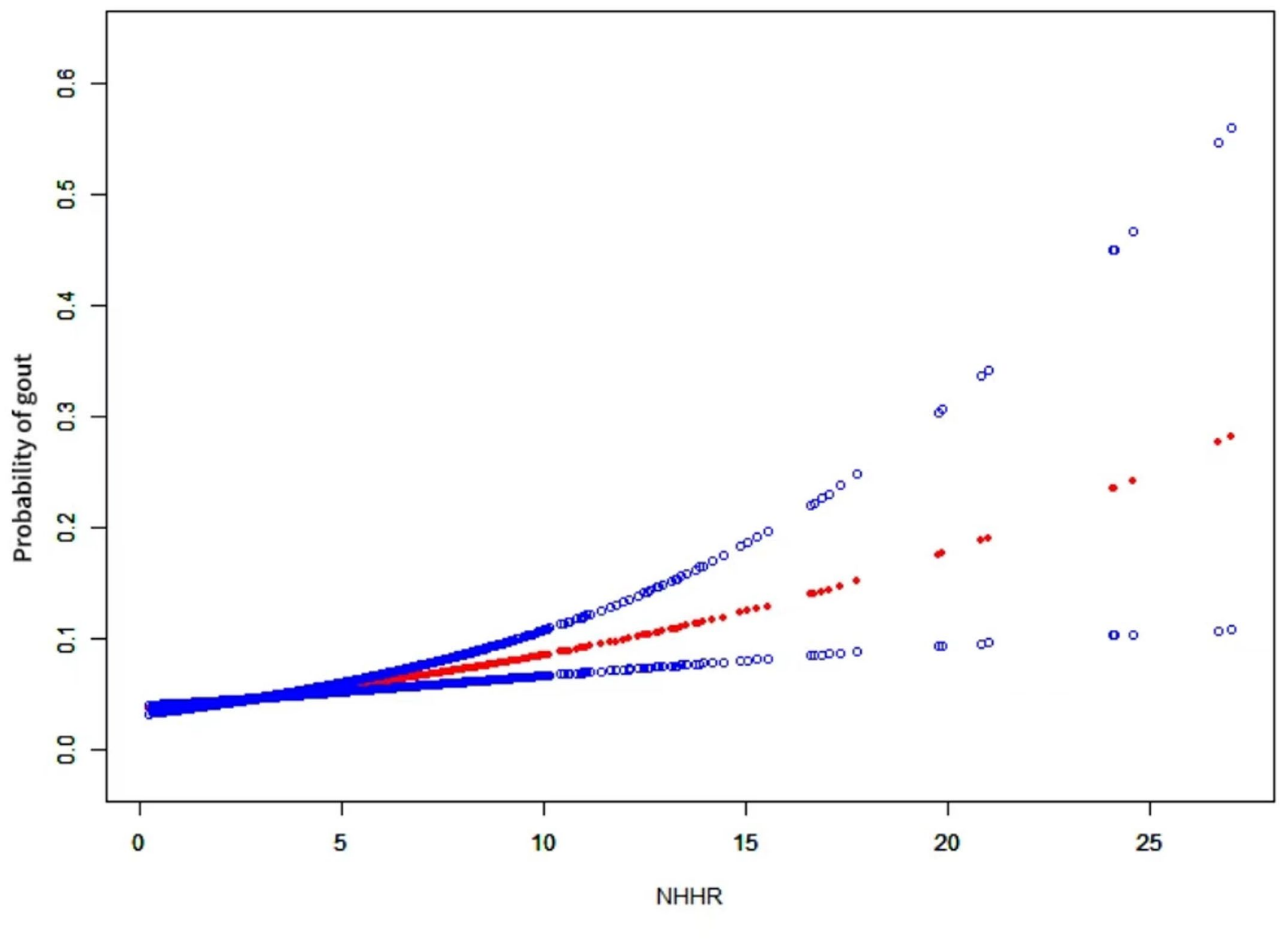
The relationship between NHHR and gout. A positive relationship between NHHR and gout was established using a generalized additive model (GAM). The solid red line signifies the smooth curve fit between variables. The blue bands represent the 95% confidence interval derived from the fit. The model was adjusted for sex, age, diabetes, race, education level, smoking habits, marital status, PIR, alcohol consumption, hypertension, eGFR, and lipid-lowering therapy.

We employed generalized additive modeling (GAM) and smoothed curve fitting to investigate the relationship between the NHHR and the probability of gout (Figure 2). Adjustments were made for the following co-variables: sex, age, diabetes, race, education level, smoking habits, marital status, PIR, alcohol consumption, hypertension, eGFR, and lipid-lowering therapy. These results confirmed the positive association between NHHR and gout development.

### 3.4 Subgroup analyses

The subgroup analysis data are presented in Table 3. The findings indicated that age and sex modified the association between the NHHR and gout (*p* < 0.05), whereas race, education level, marital status, PIR, diabetes, hypertension, eGFR, lipid-lowering therapy, and smoking habits did not affect this association. The *p*-values for the interaction of these variables (all *p* > 0.05) suggested that the likelihood of an increase in the prevalence of NHHR with gout was consistent across subgroups, except for age and sex.

**Table 3:** Subgroup analysis of the association between NHHR and gout.

NHHR	Number of gout (%), total	OR (95%CI)	*p*-value	*p* for interaction
Age (y)				0.001
<40	89 (0.82%), 10,805	1.21 (1.13, 1.30)	<0.001	
40–60	421 (4.13%), 10,189	1.09 (1.00, 1.19)	0.046	
≥60	916 (9.65%), 9,488	1.03 (0.96, 1.10)	0.412	
Sex				0.030
Male	992 (6.72%), 14,764	1.07 (1.01, 1.13)	0.037	
Female	434 (2.76%), 15,718	1.19 (1.09, 1.30)	<0.001	
Race				0.075
Mexican American	101 (2.16%), 4,671	1.16 (1.05, 1.27)	0.003	
Other Hispanic	88 (2.74%), 3,217	1.01 (0.7, 1.18)	0.896	
Non-Hispanic White	709 (5.63%), 12,600	1.12 (1.05, 1.20)	0.001	
Non-Hispanic Black	362 (5.79%), 6,252	0.99 (0.90, 1.10)	0.911	
Other races	166 (4.44%), 3,742	1.05 (0.96, 1.16)	0.298	
Education level				0.494
Less than 9th grade	163 (5.03%), 3,243	1.05 (0.92, 1.19)	0. 513	
9-11th grade	206 (4.84%), 4,257	1.15 (1.05, 1.25)	0.004	
High school graduate/GED	351 (5.05%), 6,944	1.05 (0.96, 1.15)	0.259	
Some college or AA degree	416 (4.62%), 8,995	1.14 (1.06, 1.24)	0.002	
College graduate or above	290 (4.12%), 7,043	1.07 (0.92, 1.23)	0.393	
Marital status				0.217
Married	858 (5.47%), 15,683	1.14 (1.07, 1.21)	<0.001	
Widowed	186 (7.76%), 2,397	1.11 (0.96, 1.28)	0.171	
Divorced	183 (5.54%), 3,305	0.98 (0.84, 1.13)	0.738	
Separated	47 (4.52%), 1,040	0.93 (0.79, 1.11)	0.430	
Never married	98 (1.76%), 5,584	1.12 (0.95, 1.32)	0.171	
Living with partner	54 (2.18%), 2,473	1.11 (0.98, 1.24)	0.104	
PIR				0.736
<1	275 (4.51%), 6,101	1.11 (1.00, 1.23)	0.046	
1–3	688 (4.74%), 14,506	1.08 (1.01, 1.16)	0.023	
≥3	463 (4.69%), 9,875	1.12 (1.04, 1.21)	0.005	
Smoking status				0.810
No	599 (3.51%), 17,064	1.02 (0.92, 1.13)	0.7274	
Yes	827 (6.16%), 13,418	1.11 (1.04, 1.18)	0.003	
Hypertension				0.938
No	394 (2.01%), 19,593	1.04 (0.94, 1.15)	0.4482	
Yes	1,032 (9.48%), 10,889	1.10 (1.04, 1.17)	0.002	
Diabetes				0.763
No	950 (3.61%), 26,339	1.11 (1.04, 1.18)	0.002	
Yes	476 (11.49%), 4,143	1.09 (1.00, 1.19)	0.069	
Lipid-lowering therapy				0.350
No	155 (9.33%), 1,662	1.01 (0.88, 1.17)	0.875	
Yes	568 (9.80%), 5,796	1.15 (1.05, 1.27)	0.005	
Not recorded	703 (3.05%), 23,024	1.10 (1.03, 1.17)	0.004	
eGFR				0.784
<30	52 (17.63%), 295	1.16 (0.90, 1.49)	0.256	
30–45	111 (12.94%), 858	1.15 (0.93, 1.43)	0.210	
45–60	194 (10.46%), 1,855	1.09 (0.93, 1.28)	0.311	
60–90	414 (6.13%), 6,753	1.03 (0.90, 1.18)	0.704	
≥90	655 (3.16%), 20,721	1.12 (1.06, 1.18)	<0.001	

## 4 Discussion

This cross-sectional study of 30,482 people from the United States examined the connection between the NHHR and the prevalence of gout in a sample typical of the entire country. Our findings indicate that the NHHR was positively associated with increased gout prevalence in model 3. Additional subgroup analyses provided further evidence for the consistent nature of this positive correlation. Crucially, the association remained unaffected by factors such as race, education level, marital status, PIR, BMI, smoking habits, hypertension, or diabetes. Furthermore, the relationship between the NHHR and gout prevalence was affected by age and sex. As the NHHR increases, female people under the age of 40 are more likely to develop gout. Since alcohol consumption is known to be a strong risk factor for gout, we conducted a sensitivity analysis to determine whether alcohol consumption could significantly influence our findings. The results demonstrated that excluding alcohol consumption did not substantially affect the core results, indicating that the relationship between NHHR and gout is robust regardless of alcohol consumption. Moreover, the smoothed curve fitting analysis revealed a positive relationship between the NHHR and the occurrence of gout. Therefore, the NHHR can function as an early predictor of developing gout, which is essential for the prompt prevention, diagnosis, and treatment of patients at high probability.

We further examined the relationship between sex, NHHR, and gout; the association was more significant in females. This outcome aligns with clinical observations of a positive association between SUA and TG levels in females (23). Additionally, TG and TC/HDL cholesterol levels were independently associated with SUA levels in females. In contrast, no significant association was found between SUA and lipid levels in males (24). The influence of sex on the relationship between the NHHR and gout prevalence represents a novel finding that warrants validation in future clinical research. Moreover, the more pronounced relationship observed in females should be further substantiated through a subgroup analysis.

In the subgroup analysis, variations in the NHHR were identified as significant factors (*p* < 0.05) for gout in individuals aged <40 years, which aligns with the core findings. The literature supports that age correlate not only with gout prevalence (25) but also with lipid levels (26), thereby influencing the relationship between the NHHR and gout.

Notably, this article includes an initial investigation into the association between the NHHR and gout. Emerging evidence suggests that the NHHR is an accurate indicator of the probability of lipid-related diseases (27). Nevertheless, research specifically examining the influence of the NHHR on gout is lacking, despite a significant number of studies exploring the relationship between other lipid-related factors and gout. Liang et al. conducted a cross-sectional study involving 653 patients with gout and discovered a notable association between SUA and TG levels in females (*p* = 0.039). Additionally, females had higher HDL-C levels than males (*p* < 0.05, 23). The Dietary Intervention Randomized Controlled Trial (DIRECT) conducted with 235 participants indicated a potential link between higher HDL-C levels and a lower prevalence of gout. Additionally, an improved ratio of TC to HDL-C has been linked to a reduced prevalence of gout (28). A Mendelian randomization study based on publicly available GWAS-pooled statistics demonstrated that lower HDL levels are positively correlated with a higher relationship of gout and higher SUA concentrations. Specifically, the data showed that a one standard deviation (SD) increase in HDL (~12.26 mg/dL) is associated approximately a 25% reduction in the prevalence of gout and a decrease in SUA levels of 0.09 mg/dL. The high TG levels were directly related to high uric acid levels in the blood. The findings showed that for every one SD increase in TG (approximately 112.33 mg/dL), the SUA concentrations increased by 0.10 mg/dL (29). Although these findings do not provide direct evidence of an association between the NHHR and gout, they indirectly support a positive relationship between the two. This study contributes to the research on the relationship between lipid metabolism and gout onset by utilizing innovative lipid profiles.

However, the biological mechanisms by which the NHHR influences gout development remain unclear. Existing research suggests that CLNK and WDR1 polymorphisms significantly contribute to gout in certain populations by affecting lipid metabolism. Specifically, WDR1 appears to affect gout development via the MAPK signaling pathway, whereas CLNK may operate through the STAT signaling pathway (30). Emerging evidence has highlighted the role of aldosterone in uric acid metabolism, particularly among hypertensive patients. Song et al. explored the impact of elevated plasma aldosterone concentrations on the risk of hyperuricemia and gout, finding a significant dose–response relationship. Their study demonstrated that plasma aldosterone levels above 14 ng/dL were strongly associated with an increased risk of developing hyperuricemia and gout. This further supports the hypothesis that hormonal regulation, particularly aldosterone, should be considered when assessing the risk and pathophysiology of gout in hypertensive populations (31). Additionally, recent evidence underscores the role of environmental factors, such as phthalate exposure, in kidney-related conditions. Di et al. (32) found that elevated urinary levels of phthalate metabolites, particularly MiBP and MBzP, were associated with an increased risk of nephrolithiasis, especially among individuals with higher serum calcium levels, suggesting a potential interaction between phthalate exposure and calcium metabolism. These findings emphasize the importance of considering environmental exposures within the context of metabolic dysregulation, which may be relevant to gout and other conditions involving altered purine metabolism (32). Moreover, recent studies have highlighted the role of systemic inflammatory markers in the development of metabolic conditions. For instance, Di et al. (33) found that the systemic immune-inflammation index (SII), derived from neutrophil, lymphocyte, and platelet counts, is significantly associated with an increased risk of kidney stones, especially among adults under 50 years old. This underscores the importance of systemic inflammation in disease processes such as gout and suggests a potential parallel in the metabolic and inflammatory pathways involved in both conditions (33). Additionally, a study involving mice demonstrated that HDL can modulate gout by interacting with synovial tissue and fluid cells (34). However, the precise mechanisms remain to be elucidated through further clinical studies. Nonetheless, maintaining low NHHR levels is crucial for reducing the prevalence of gout.

NHHR, a novel composite indicator of atherogenic lipid composition (6), is superior to conventional single-lipid variables in evaluating the extent of atherosclerosis (35). It also serves as an important lipid marker for preventing plaque formation (36). Additionally, findings from the NAGALA longitudinal cohort study conducted in Japan indicate that the NHHR is a more effective indicator than traditional lipid markers for forecasting the likelihood of developing diabetes (6). Lin et al. (37) conducted a study of 9,764 Chinese volunteers and discovered that the NHHR was a more powerful indicator of insulin resistance and diabetes than other traditional lipid markers. The diagnostic value of the NHHR surpasses that of conventional lipid indicators in predicting non-alcoholic fatty liver disease (NAFLD) (38) and metabolic syndrome (13). In conclusion, the NHHR has demonstrated excellent predictive ability in several studies and therefore has great potential for extensive clinical applications as a widely used biomarker.

### 4.1 Study strengths and limitations

This study had several merits. First, the study had a substantial sample size of 30,482 participants. Second, confounding variables were adjusted to ensure reliable results. Third, subgroup examinations were conducted to assess the strength and reliability of the association between the NHHR and odds of developing gout in various groups. Association between the NHHR and gout. However, this study had certain limitations. First, because of its cross-sectional design, the study was unable to establish a causal relationship between the NHHR and gout. Second, although multiple co-variables were considered, excluding all potential factors that might influence the study results was not feasible. Furthermore, we used the E-value sensitivity analysis to quantify the potential implications of unmeasured confounders and found that an unmeasured confounder was unlikely to explain the entirety of the treatment effect. Third, the observed differences in age and sex among the participants indicate that future research should employ larger and more homogeneous samples to control for these variables. Forth, we acknowledge that the handling of missing data can influence the results of the analysis. In this study, we addressed missing data by employing dummy variables for lipid-lowering therapy, where a large proportion of data was missing, and median interpolation for covariates with fewer missing values. These methods help mitigate potential biases introduced by missing data, ensuring that the core associations between NHHR and gout remain robust despite missing information. Finally, the design of the database may have introduced a selection bias, potentially affecting the results.

## 5 Conclusion

The data clearly showed a significant association between higher NHHR scores and the probability of gout in individuals in the United States. This highlights the need to identify individuals at prevalence of gout by monitoring aberrant NHHR levels. Although the cross-sectional design of this study hinders the ability to make causal inferences, it offers a foundation for future prospective investigations to confirm the causal association between the NHHR and gout.

## Data Availability

The original contributions presented in the study are included in the article/Supplementary material, further inquiries can be directed to the corresponding author.
