# Supplementary material for: Association of non-high-density lipoprotein cholesterol-to-high-density lipoprotein cholesterol ratio (NHHR) with gout prevalence: a cross-sectional study
Source: Front Nutr. 2024 Oct 24;11:1480689. doi: 10.3389/fnut.2024.1480689 (PMC11541233; doi:10.3389/fnut.2024.1480689)
Supplement: Supplementary file 1 [file Table_1.DOCX]

Supplementary Table 1. Summary of Missing Data and Handling Methods for Covariates.

| **Covariables** | **The number of missing values** | **Proportion missing (%)** |
| --- | --- | --- |
| Lipid-lowering therapy | 23015 | 75.50 |
| Drinking | 11672 | 38.29 |
| BMI | 375 | 1.23 |
| PIR | 2846 | 0.34 |
| SUA level | 112 | 0.37 |
| eGFR | 431 | 1.41 |
