# Supplementary material for: Association of non-high-density lipoprotein cholesterol-to-high-density lipoprotein cholesterol ratio (NHHR) with gout prevalence: a cross-sectional study
Source: Front Nutr. 2024 Oct 24;11:1480689. doi: 10.3389/fnut.2024.1480689 (PMC11541233; doi:10.3389/fnut.2024.1480689)
Supplement: Supplementary file 2 [file Table_2.DOCX]

Supplementary table 2. Sensitivity Analysis Results for the Association Between NHHR and Gout, Excluding Alcohol Consumers.

| Exposure | Model 1 | Model 2 | Model 3 |
| --- | --- | --- | --- |
|  | OR (95%CI), p-value | OR (95%CI), p-value | OR (95%CI), p-value |
| Continuous | 1.13 (1.08, 1.18), <0.001 | 1.15 (1.01, 1.21), <0.001 | 1.10 (1.05, 1.16), <0.001 |
| NHHR quartile |  |  |  |
| Q1 | Reference | Reference | Reference |
| Q2 | 1.13 (0.91, 1.40), 0.283 | 1.08 (0.86, 1.36), 0.498 | 1.03 (0.82, 1.30), 0.790 |
| Q3 | 1.21 (0.95, 1.53), 0.120 | 1.16 (0.91, 1.49), 0.242 | 1.04 (0.81, 1.34), 0.744 |
| Q4 | 1.61 (1.29, 2.01), <0.001 | 1.62 (1.28, 2.05), <0.001 | 1.34 (1.05, 1.71), 0.022 |
| p for trend | <0.001 | <0.001 | 0.031 |
| Male |  |  |  |
| Continuous | 1.01 (0.95, 1.08), 0.761 | 1.11 (1.05, 1.17), <0.001 | 1.07 (1.01, 1.13), 0.036 |
| NHHR quartile |  |  |  |
| Q1 | Reference | Reference | Reference |
| Q2 | 0.83 (0.63, 1.10), 0.198 | 0.91 (0.69, 1.21), 0.523 | 0.87 (0.66, 1.14), 0.318 |
| Q3 | 0.79 (0.58, 1.06), 0.122 | 0.99 (0.72, 1.34), 0.930 | 0.89 (0.65, 1.22), 0.470 |
| Q4 | 0.86 (0.66, 1.12), 0.272 | 1.26 (0.96, 1.67), 0.103 | 1.06 (0.80, 1.40), 0.687 |
| p for trend | 0.377 | 0.064 | 0.527 |
| Female |  |  |  |
| Continuous | 1.26 (1.15, 1.37), <0.001 | 1.27 (1.16, 1.39), <0.001 | 1.19 (1.09, 1.30), <0.001 |
| NHHR quartile |  |  |  |
| Q1 | Reference | Reference | Reference |
| Q2 | 1.40 (0.93, 2.11), 0.107 | 1.38 (0.91, 2.09), 0.133 | 1.32 (0.86, 2.01), 0.208 |
| Q3 | 1.46 (1.03, 2.07), 0.039 | 1.46 (1.02, 2.09), 0.043 | 1.29 (0.89, 1.86), 0.181 |
| Q4 | 2.52 (1.75, 3.64), <0.001 | 2.57 (1.75, 3.76), <0.001 | 2.05 (1.39, 3.03), <0.001 |
| p for trend | <0.001 | <0.001 | 0.001 |

Notes: Model 1: unadjusted. Model 2: Adjusted for sex, age, and race. Model 3: Adjusted for sex, age, race, education level, smoking, marital status, PIR, diabetes, hypertension, eGFR, and lipid-lowering therapy. The stratified analysis was not adjusted for sex.
