# Supplementary material for: Association of non-high-density lipoprotein cholesterol-to-high-density lipoprotein cholesterol ratio (NHHR) with gout prevalence: a cross-sectional study
Source: Front Nutr. 2024 Oct 24;11:1480689. doi: 10.3389/fnut.2024.1480689 (PMC11541233; doi:10.3389/fnut.2024.1480689)
Supplement: Supplementary file 3 [file Table_3.DOCX]

Supplementary Table 3. Collinearity Assessment of Covariates Using Variance Inflation Factors (VIF)

| covariates | variance inflation factors (VIF) |
| --- | --- |
| Drinking | 1 |
| BMI | 2.4 |
| Hypertension | 1.4 |
| Lipid-lowering therapy | 1.4 |
| Race | 1.1 |
| Sex | 1.4 |
| Age | 2.8 |
| Education level | 1.4 |
| Marital status | 1.2 |
| PIR | 1.3 |
| Diabetes | 1.2 |
| Smoking | 1.1 |
| Serum uric acid level | 1.4 |
| Gout | 1.1 |
| eGFR | 3.4 |
